# Enhancing clinical competency in infectious disease training: a longitudinal study of Mini-CEX implementation for medical interns

**DOI:** 10.3389/fmed.2025.1582218

**Published:** 2025-07-09

**Authors:** Xiaohao Wang, Hu Li, Yi Liu, Dazhi Zhang, Dachuan Cai, Shan Zhong

**Affiliations:** Department of Infectious Diseases, The Second Affiliated Hospital of Chongqing Medical University, Chongqing, China

**Keywords:** Mini-CEX, clinical skills assessment, infectious disease department, interns, medical education

## Abstract

**Objective:**

This study aimed to evaluate the effectiveness of the Mini-Clinical Evaluation Exercise (Mini-CEX) in assessing and improving clinical competencies among medical interns during a 4-week infectious disease rotation.

**Methods:**

Forty-six medical interns were assessed using Mini-CEX at the start and end of their rotation. The tool evaluated seven domains: history taking, physical examination, clinical judgment, humanistic care, communication skills, organizational effectiveness, and overall competence. Teaching physicians were trained uniformly before the trial. After the internship, interns and teaching physicians completed questionnaires and interviews.

**Results:**

During the internship period when Mini-CEX was implemented, significant improvements were observed in interns’ clinical skills across multiple domains. For example, the average scores of interns in history taking increased from 5.12 ± 0.89 to 6.22 ± 1.01, and in physical examination from 3.97 ± 0.69 to 5.24 ± 0.86. Interns showed high acceptance and satisfaction with Mini-CEX. The implementation of Mini-CEX also improved teaching effectiveness, with enhanced teacher-student interactions.

**Conclusion:**

Mini-CEX is a feasible and effective tool for clinical skill development in infectious disease training. Its structured feedback mechanism aligns with competency-based medical education (CBME) goals. Future studies should explore its scalability across disciplines and integration with complementary assessment tools.

## Introduction

Infectious disease management demands rapid clinical judgment, rigorous infection control, and compassionate patient care. Medical interns rotating through this specialty face unique challenges, including exposure to diverse pathogens and complex cases, necessitating robust training and assessment frameworks (1). Competency-Based Medical Education (CBME) emphasizes formative assessments to bridge theory and practice (2), yet effective tools tailored to infectious disease training remain underexplored in China’s evolving medical education landscape.

Globally, the Mini-Clinical Evaluation Exercise (Mini-CEX)—a workplace-based assessment tool offering direct observation and immediate feedback—has demonstrated efficacy in enhancing clinical reasoning and communication skills across specialties such as internal medicine (3) and pediatrics (4). However, its application in high-risk environments like infectious disease departments, where diagnostic precision and biosafety are paramount, remains limited. This gap is particularly pronounced in China, where systemic challenges intersect with the urgent need to modernize medical education.

China’s medical education system has historically prioritized theoretical knowledge over hands-on clinical training (5). While recent reforms under initiatives like Healthy China 2030 emphasize competency-based approaches to address workforce shortages in critical specialties (6), significant barriers persist. The COVID-19 pandemic underscored the urgency of training clinicians capable of managing outbreaks with both technical proficiency and ethical sensitivity (7). Yet, resistance to abandoning traditional exam-centric evaluations and limited faculty training in modern assessment methods hinder progress (8).

Within this context, infectious disease training faces unique pressures. The discipline requires not only mastery of complex pathophysiology but also adherence to stringent biosafety protocols—skills poorly assessed by conventional written exams. Furthermore, China’s vast geographic and socioeconomic disparities mean rural interns often encounter resource-limited settings ill-aligned with urban-centric training models (9). These challenges highlight the need for adaptable, context-sensitive tools like Mini-CEX, which can standardize assessments while accommodating regional variability.

This study investigates Mini-CEX’s role in addressing these challenges. By evaluating its impact on intern skill development within a high-risk clinical setting, we aim to contribute actionable insights for aligning China’s CBME reforms with global best practices.

## Methods

### Study design and participants

A longitudinal study was conducted with 46 medical interns during a 4-week infectious disease rotation at The Second Affiliated Hospital of Chongqing Medical University (February 2023–January 2024). Participants represented 80% of eligible interns, with exclusion criteria including prior infectious disease training or incomplete rotations. To standardize clinical exposure, interns followed a structured curriculum: (1) Weekly case discussions: Focused on viral hepatitis, tuberculosis, HIV, and emerging pathogens; and (2) Clinical practice: Minimum of 10 patient encounters per week, supervised by attending physicians.

The sample size was calculated *a priori* using GPower 3.1. For paired *t*-tests (two-tailed, *α* = 0.05, power [1−*β*] = 0.80), an effect size of d = 0.5 [based on prior Mini-CEX studies (3, 10)] required 34 participants. Accounting for 20% attrition, 46 interns were recruited. This exceeds the minimum requirement and aligns with similar longitudinal assessments. The exclusion criteria were previous infectious diseases training or incomplete rotation. Referring to similar studies (10), this sample size is sufficient to detect significant changes in clinical skills scores. The study analyzed through paired *t*-tests.

### Ethical considerations

The study was approved by the hospital’s Ethics Committee (No. 2023-ID-015). Written informed consent was obtained, emphasizing voluntary participation and data anonymity.

### Intervention

#### Mini-CEX implementation

Assessment Tool: A modified Mini-CEX rubric (Supplementary Table 1) evaluated seven domains using a 9-point Likert scale: 1–3: Needs improvement; 4–6: Competent; 7–9: Exemplary. The rubric was tailored to emphasize: (1) Biosafety protocols: Proper use of personal protective equipment (PPE); (2) Epidemiological history: Detailed travel and contact histories; and (3) Rapid decision-making: Prioritizing differential diagnoses in febrile patients. The Mini-CEX rubric embedded biosafety protocols (e.g., PPE use) within the “Organizational Effectiveness” domain and epidemiological history-taking within “History Taking” to align with infectious disease-specific competencies.

The improved Mini-CEX integrates the core capabilities of infectious disease management into each assessment dimension: (1) The clinical judgment dimension focuses on assessing the differential diagnosis of infectious diseases (such as viral hepatitis vs.) Bacterial infections and treatment decisions; (2) Evaluate the implementation of infection control measures (such as standard prophylaxis and contact isolation) in the organizational effectiveness dimension; and (3) Medical history collection emphasizes the completeness of epidemiological history (such as exposure to epidemic areas and occupational risks).

Validation of the Modified Mini-CEX Tool: Before implementation, the modified rubric underwent rigorous validation. First, content validity was established through expert consensus by four senior infectious disease physicians (>5 years of experience), who aligned domains (e.g., biosafety protocols, epidemiological history-taking) with China’s Infectious Disease Competency Framework. Second, inter-rater reliability (IRR) was tested during pilot assessments: four assessors independently evaluated 10 standardized patient scenarios. IRR analysis showed substantial agreement (*κ* = 0.82, 95% CI: 0.75–0.89). Third, structural validity was confirmed via factor analysis (Cronbach’s *α* = 0.91 for all domains).

### Procedure

This was a longitudinal pre-post interventional study evaluating clinical skills changes over a 4-week rotation. The assessment is conducted in a real clinical environment. Interns need to complete the diagnosis and treatment of at least 10 real patients per week, and the supervising doctor conducts the assessment through bedside observation.

Pre-rotation assessment: Conducted during the first 3 days of the rotation.
Post-rotation assessment: Completed in the final 3 days.
Feedback session: 10 min debriefing after each assessment, focusing on actionable steps for improvement.

#### Assessor training

Four attending physicians with ≥5 years of infectious disease experience underwent:

**Simulation workshops:** Standardized patient scenarios (e.g., febrile patient with unknown etiology).
**Inter-rater reliability testing:** *κ* = 0.82 after evaluating 10 pilot cases.
**Biannual refresher courses:** To maintain consistency in evaluations.

### Data collection

**Quantitative:** Pre- and post-rotation Mini-CEX scores.
**Intern surveys:** 15-item questionnaire assessing Mini-CEX acceptability (Supplementary Table 2).
**Supervisor feedback:** 10-item survey on teaching experiences (Supplementary Table 3).

### Statistical analysis

SPSS 21.0 analyzed data using paired *t*-tests (pre-post comparisons) and descriptive statistics (survey responses). Significance was set at *p* < 0.05.

## Results

### Improvement in clinical skills of interns

The following table summarizes the comparison of interns’ Mini-CEX scores at the beginning and end of their rotations. Results demonstrated heterogeneous improvements across domains. Organizational effectiveness (38.62% increase) and physical examination (31.99%) showed the steepest gains, likely driven by structured training in infection control protocols. In contrast, clinical judgment (14.68%) exhibited more modest improvement, indicating a need for targeted case-based reasoning workshops (Table 1).

**Table 1:** Comparison of interns Mini-CEX scores at the beginning and end of their rotations.

Assessment area	Beginning scores (Mean ± SD)	End scores (Mean ± SD)	Percentage improvement (%)	*p*-value
History taking	5.12 ± 0.89	6.22 ± 1.01	21.48	<0.001
Physical examination	3.97 ± 0.69	5.24 ± 0.86	31.99	<0.001
Clinical judgment	5.45 ± 0.79	6.25 ± 0.98	14.68	<0.001
Humanistic care	4.85 ± 0.68	5.98 ± 0.76	23.29	<0.001
Communication skills	4.78 ± 0.65	6.05 ± 0.86	26.57	<0.001
Organizational effectiveness	4.22 ± 0.58	5.85 ± 0.70	38.62	<0.001
Overall competence	4.08 ± 0.51	5.21 ± 0.84	27.69	<0.001

These data highlight the changes in interns’ clinical skills as measured by the Mini-CEX evaluation system.

### Acceptance and satisfaction of Mini-CEX among interns

Our survey and interviews revealed that interns had a high level of acceptance and satisfaction with the Mini-Clinical Evaluation Exercise (Mini-CEX) assessment system. Specifically, 80.43% of interns understood the purpose of Mini-CEX assessment. 71.74% believed that Mini-CEX assessment aided them in promptly identifying their deficiencies in clinical skills.

In terms of assessment administration, 60.87% of interns perceived that the supervisors took the assessment seriously, 56.52% deemed the allocated time for the assessment as reasonable, and 66.22% of interns considered the assessment process to be non-disruptive to their clinical internship work. 76.09% of interns demonstrated a proactive approach by carefully considering the feedback from supervisors and working diligently to address their deficiencies.

Regarding the assessment’s alignment with practical clinical scenarios, 54.35% of interns opined that Mini-CEX was more relevant than traditional assessment methods, and 63.04% acknowledged the fairness and objectivity of Mini-CEX assessment.

Qualitative analysis identified three key themes from intern interviews: perceived feedback timeliness (82% mentioned “immediate corrections”), assessment fairness (63% valued rubric objectivity), and cultural congruence (71% felt feedback aligned with educational norms). The Table 2 summarizes the interns’ overall evaluation of Mini-CEX.

**Table 2:** Overall evaluation of interns towards Mini-CEX (*n* = 46).

Evaluation item	Yes	No	Uncertain
Understand the purpose	37 (80.43)	4 (8.69)	5 (1.87)
Beneficial to learning	33 (71.74)	2 (4.25)	11 (23.91)
Supervisor takes it seriously	28 (60.87)	8 (17.39)	10 (21.74)
Reasonable assessment time	26 (56.52)	10 (21.74)	10 (21.74)
Does not hinder internship	30 (66.22)	7 (15.22)	9 (19.56)
Takes teacher feedback seriously	35 (76.09)	3 (6.52)	8 (17.39)
Reflects real-world competence	25 (54.35)	12 (26.09)	9 (19.56)
Objective and fair results	29 (63.04)	5 (1.87)	12 (26.09)

The above data present the interns’ responses and attitudes towards the Mini-CEX assessment system.

### Improvement in teaching effectiveness

The implementation of the Mini-Clinical Evaluation Exercise (Mini-CEX) assessment system led to improvements in teaching effectiveness. While 100% of supervisors endorsed Mini-CEX’s educational value, 75% reported time constraints as a burden, noting that “integrating assessments into daily rounds required extra effort.” Only 25% felt the tool did not impose additional workload. Supervisors highlighted enhanced teacher-student interaction but noted time constraints as a primary barrier (75% reported “workload challenges”). The following table presents the overall evaluation of supervisors towards Mini-CEX (Table 3).

**Table 3:** Overall evaluation of supervisors towards Mini-CEX (*n* = 4).

Evaluation item	Yes	No	Uncertain
Enhanced teaching effectiveness	4 (100)	0 (0)	0 (0)
Takes it seriously	4 (100)	0 (0)	0 (0)
Convenient to implement	3 (75)	0 (0)	1 (25)
Received relevant training	4 (100)	0 (0)	0 (0)
Students take it seriously	3 (75)	0 (0)	1 (25)
Does not impose additional burden	1 (25)	3 (75)	0 (0)
Enhances teaching achievement	3 (75)	1 (25)	0 (0)
Promotes teacher-student interaction	4 (100)	0 (0)	0 (0)

These findings illustrate the impact of the Mini-CEX system on teaching from the perspective of the supervising physicians.

## Discussion

The findings of this study underscore the efficacy of Mini-CEX as a formative assessment tool in infectious disease training, aligning with global trends in competency-based medical education (CBME). Improvements in infection control-related skills (e.g., PPE compliance) were reflected in the Organizational Effectiveness domain (38.62% increase), while epidemiological history-taking precision was subsumed under History Taking (21.48% improvement). These results resonate with prior studies demonstrating Mini-CEX’s utility in enhancing procedural skills and situational awareness in specialties such as emergency medicine (11) and anesthesiology (12). However, the magnitude of improvement in infectious disease-specific competencies, such as meticulous PPE use and epidemiological history-taking, exceeds outcomes reported in general internal medicine settings (3), suggesting that context-specific adaptations of Mini-CEX amplify its impact.

The pronounced gains in physical examination skills may reflect the unique demands of infectious disease management, where systematic evaluation of rashes, lymphadenopathy, or respiratory signs is critical. This aligns with findings by Kurdi et al. (13), who noted that Mini-CEX’s structured feedback improves technique standardization. Similarly, the marked improvement in organizational effectiveness-a domain encompassing infection control protocols and interdisciplinary coordination-echoes studies in outbreak settings where structured workflows reduce transmission risks (7). These results collectively validate Mini-CEX’s role in fostering both technical and operational competencies, which are indispensable in pandemic preparedness (14).

Intern perceptions of Mini-CEX revealed a paradox: while 76.1% valued feedback, only 54.3% viewed it as more clinically relevant than traditional assessments. This discrepancy may stem from the tension between Mini-CEX’s simulated scenarios and the unpredictability of real-world infectious disease cases, a challenge also observed in Pakistani medical schools (15). To bridge this gap, future implementations could integrate dynamic simulations of emerging pathogens (e.g., COVID-19 or avian influenza), mirroring strategies successfully employed in telemedicine training (16). Furthermore, the cultural context of feedback acceptance-76.1% of interns actively addressed supervisors’ input—may reflect Confucian educational values emphasizing humility and incremental improvement (17), a factor less pronounced in Western studies (18).

Supervisors unanimously endorsed Mini-CEX’s capacity to enhance teacher-student interaction, corroborating its role as a catalyst for mentored learning (19). However, 75% reported time constraints, a barrier consistent with faculty experiences in neurology training (20). To mitigate this, institutions could adopt a “distributed assessment” model, embedding Mini-CEX into routine clinical activities rather than isolating it as a standalone exercise. Training senior residents as assessors, as proposed by Al Ansari et al. (21), may further alleviate faculty workload while promoting peer-to-peer learning.

### Limitations and future directions

The small sample size (*n* = 46) limits the statistical power and generalizability of findings, particularly to resource-constrained or rural settings. The absence of a control group precludes definitive attribution of skill improvements solely to Mini-CEX. Natural progression during rotations or concurrent educational activities (e.g., case discussions) may have confounded results, a limitation also noted in Brazilian Mini-CEX trials (10). Additionally, the single-center design and small sample size limit generalizability to rural or resource-limited settings, where infectious disease burdens are often highest (9). Future multi-center studies should employ mixed-methods designs to explore contextual variability, particularly in regions with disparate healthcare infrastructures.

Technological integration presents another promising avenue (22). Mobile-based Mini-CEX platforms, as piloted in German clerkships (23), could streamline data collection and enable real-time feedback, addressing supervisors’ time concerns. Combining Mini-CEX with longitudinal entrustable professional activities (EPAs) may also provide a more holistic view of competency development over time (2).

## Conclusion

This study positions Mini-CEX as a transformative tool in infectious disease education, bridging the gap between China’s CBME reforms and global pedagogical advancements. By addressing cultural, logistical, and contextual barriers, Mini-CEX can empower a new generation of clinicians to navigate the complexities of emerging infectious threats with skill and compassion, in alignment with evolving global pedagogical priorities.

## Data Availability

The datasets presented in this article are not readily available because the datasets generated and/or analyzed during the current study are not publicly available due to privacy concerns. Requests to access the datasets should be directed to zhongshan@cqmu.edu.cn.
