# Supplementary material for: Enhancing clinical competency in infectious disease training: a longitudinal study of Mini-CEX implementation for medical interns
Source: Front Med (Lausanne). 2025 Jul 9;12:1582218. doi: 10.3389/fmed.2025.1582218 (PMC12283664; doi:10.3389/fmed.2025.1582218)
Supplement: Supplementary file 1 [file Table_1.doc]

Supplementary Table 1

**Mini-Clinical Evaluation Exercise（Mini-CEX）**

**For Intern of Infectious Department**

(Before entering the department □/ After leaving the department □）

Teacher： 　Date：________________

Intern：

Diagnosis：__________________________________________________________________

Assessment Site：□Outpatient Department □Emergency Department □Inpatient Department

Patient Age：_______　Gender：______　 □First visit　□Return visit

Disease Complexity：□Low　□Medium　□High

Key of assessment：□History taking　□Physical examination　□Clinical diagnosis

□Therapeutic schedule □Health education

|  | Need to be improved | Qualified | Excellent |
| --- | --- | --- | --- |
| 1.History Taking（□unobserved） | 1 2 3 | 4 5 6 | 7 8 9 |
| 2.Physical Examination（□unobserved） | 1 2 3 | 4 5 6 | 7 8 9 |
| 3.Clinical Judgment（□unobserved） | 1 2 3 | 4 5 6 | 7 8 9 |
| 4.Humanistic Care（□unobserved） | 1 2 3 | 4 5 6 | 7 8 9 |
| 5.Communication Skills（□unobserved） | 1 2 3 | 4 5 6 | 7 8 9 |
| 6.Organizational Effectiveness（□unobserved） | 1 2 3 | 4 5 6 | 7 8 9 |
| 7.Overall Competence（□unobserved） | 1 2 3 | 4 5 6 | 7 8 9 |

(If proportion of “Not observed” is more than 5%, extending the evaluation period (4 weeks) is needed.)

Observation time：________________minutes Feedback time：_________________minutes

Teacher satisfaction： Low 1 2 3 4 5 6 7 8 9 10 High

Intern satisfaction： Low 1 2 3 4 5 6 7 8 9 10 High

**Advice：_______________________________________________________________________________________________________________________________________________________________________________________________________________________________**

Intern signature：__________________ Teacher signature：____________________
