# Supplementary material for: Enhancing clinical competency in infectious disease training: a longitudinal study of Mini-CEX implementation for medical interns
Source: Front Med (Lausanne). 2025 Jul 9;12:1582218. doi: 10.3389/fmed.2025.1582218 (PMC12283664; doi:10.3389/fmed.2025.1582218)
Supplement: Supplementary file 2 [file Table_2.docx]

Supplementary Table 2

Questionnaire of Mini-CEX for Interns

Intern Name： Date:

1. Do you understand the purpose of Mini-CEX?

Yes□ No□ Uncertain□

1. Do you think Mini-CEX is beneficial to Learning?

Yes□ No□ Uncertain□

1. Does your supervisor take it Seriously?

Yes□ No□ Uncertain□

1. Do you think the assessment time is reasonable ?

Yes□ No□ Uncertain□

1. Do you think the procedure of Mini-CEX hinder the internship?

Yes□ No□ Uncertain□

1. Do you take the teacher’s feedback seriously?

Yes□ No□ Uncertain□

1. Do you think the result of Mini-CEX reflect your real competence?

Yes□ No□ Uncertain□

1. Do you think the result of evaluation is objective and fair ?

Yes□ No□ Uncertain□
