# Supplementary material for: Enhancing clinical competency in infectious disease training: a longitudinal study of Mini-CEX implementation for medical interns
Source: Front Med (Lausanne). 2025 Jul 9;12:1582218. doi: 10.3389/fmed.2025.1582218 (PMC12283664; doi:10.3389/fmed.2025.1582218)
Supplement: Supplementary file 3 [file Table_3.docx]

Supplementary Table 3

Questionnaire of Mini-CEX for Teacher

Teacher Name： Date:

1. Do you think the Mini-cex enhance your teaching effectiveness?

Yes□ No□ Uncertain□

1. Do you take Mini-CEX Seriously?

Yes□ No□ Uncertain□

1. Do you think it’s convenient to implement?

Yes□ No□ Uncertain□

1. Did you receive relevant training of Mini-CEX before the procedure?

Yes□ No□ Uncertain□

1. Do you think students take it seriously?

Yes□ No□ Uncertain□

1. Do you think the evaluation impose additional burden?

Yes□ No□ Uncertain□

1. Do you think Mini-CEX enhance your teaching achievement?

Yes□ No□ Uncertain□

1. Do you think Mini-CEX promotes teacher-student interaction ?

Yes□ No□ Uncertain□
